# Rapid Healing of Necrotizing Fasciitis Using the Kerecis Fish Skin Xenograft: A Clinical Case Report

**DOI:** 10.7759/cureus.73060

**Published:** 2024-11-05

**Authors:** Kevin M Posner, Cassandra Bakus, Samir Sodha

**Affiliations:** 1 Department of Orthopedic Surgery, Hackensack Meridian School of Medicine, Nutley, USA; 2 Department of Orthopedic Surgery, Hackensack University Medical Center, Hackensack, USA

**Keywords:** necrotizing fascitis, orthopedic hand surgery, wound infections, wound management, xenografts

## Abstract

This case report explores the application of the Kerecis^TM^ fish skin xenograft, derived from North Atlantic Cod, in treating a large full-thickness wound resulting from necrotizing fasciitis (NF). A 41-year-old female with multiple comorbidities presented with NF of the dorsal forearm and hand. Initially managed with serial washouts with extensive debridement, the remaining dorsal forearm full-thickness wound with exposed tendons was treated with an application of the Kerecis^TM^ xenograft. The graft facilitated healing, evidenced by rapid epithelialization and decreased pain without the use of additional skin grafting. Traditionally used for chronic conditions, the use of Kerecis^TM^ in this acute, complex wound highlights its potential for integrating into human tissue and modulating inflammation, as well as acting as an antimicrobial barrier. This case underscores the need for further research into the effectiveness of fish skin xenografts in acute and complex wounds, suggesting a potential shift in emergency wound care practices.

## Introduction

Necrotizing fasciitis (NF) is a serious and often life-threatening soft tissue infection that leads to the rapid destruction of fascia and subsequent necrosis. First described during the fifth century BCE, NF has been referred to using multiple names throughout the years: phagedena, pyoderma gangrenosum, progressive bacterial synergistic gangrene, and non-clostridial gas gangrene. 

NF can typically occur after an injury, such as a minor or blunt trauma, cut, abrasion, insect bite, or surgical site incisions. Any part of the body may be affected by NF; however, the extremities are the most common, and there is often no identifiable point of entry [1]. Any time there is a host that has an immunocompromised state, there is an increased risk for NF; however, other identified risk factors include diabetes, obesity, peripheral vascular disease, smoking, intravenous drug use, and alcohol abuse, to name a few [2,3]. The condition may begin as a small inflamed area of soft tissue or skin that quickly progresses to fasciitis [4]. The relatively benign findings in the early stages of the disease course may ultimately lead to misdiagnosis of cellulitis [4]. Classic skin findings in NF include blisters and bullae along with discoloration that then turns to necrotic sloughing tissue [4].

Early identification and surgical debridement are critical to manage this life-threatening condition. Following the removal of necrotic tissue, reconstructing the substantial soft tissue defects presents a clinical challenge. Such defects may require the use of skin flaps or split-thickness grafts, while others may require the use of free-tissue transfer [3]. One such alternative that has been introduced to treat diabetic wounds, traumatic wounds, partial-thickness burns, acute surgical incisions, and necrotic wounds is the Kerecis^TM^ fish skin xenograft products [5,6]. The Kerecis^TM^ fish skin xenograft is a product derived from minimally processed North Atlantic Cod, which allows it to act similarly to human skin. The company utilizes a proprietary, patented process that allows the preservation of its protein and matrix structure, along with its lipid composition. This porous structure is then able to allow the ingrowth of dermal cells and capillaries for healing. The process also helps to maintain long-chain omega-3 fatty acids, which helps to mitigate cytokine signaling that acts as an anti-inflammatory and provides an antimicrobial barrier. 

Kerecis^TM^ is typically used for chronic wounds, such as diabetic ulcers and burns, where it serves as a scaffold that integrates with human tissue. Despite its proven benefits in chronic and complex wounds, the literature lacks examples of Kerecis^TM^ being utilized for acute wounds resulting from conditions like NF. This case report aims to address this gap by detailing the use of Kerecis^TM^ in the treatment of a large full-thickness wound with exposed extensor tendons that remained after the treatment of NF.

## Case presentation

A 41-year-old female with a past medical history significant for end-stage renal disease on hemodialysis, hypertension, and hypothyroidism was diagnosed with an acute episode of NF affecting the same extremity as her arteriovenous (AV) fistula in the antecubital fossa. She underwent wide debridement of the infected fascia and washout on September 28, 2023. The full-thickness skin and fascia deficit with exposed extensor tendons on the ulnar side of the wound required surgical intervention, but the presence of the AV fistula on the same extremity in close proximity to the wound precluded the use of rotational or free flaps due to the increased risk of complications. The elevated INR and exposed extensor tendons made primary skin grafting impractical, as it required a well-prepared wound bed with adequate vascularization. Kerecis^TM^ was selected as a less invasive option, providing a scaffold for vascular ingrowth and facilitating healing without the risks associated with more invasive surgical techniques.

**Figure 1:**
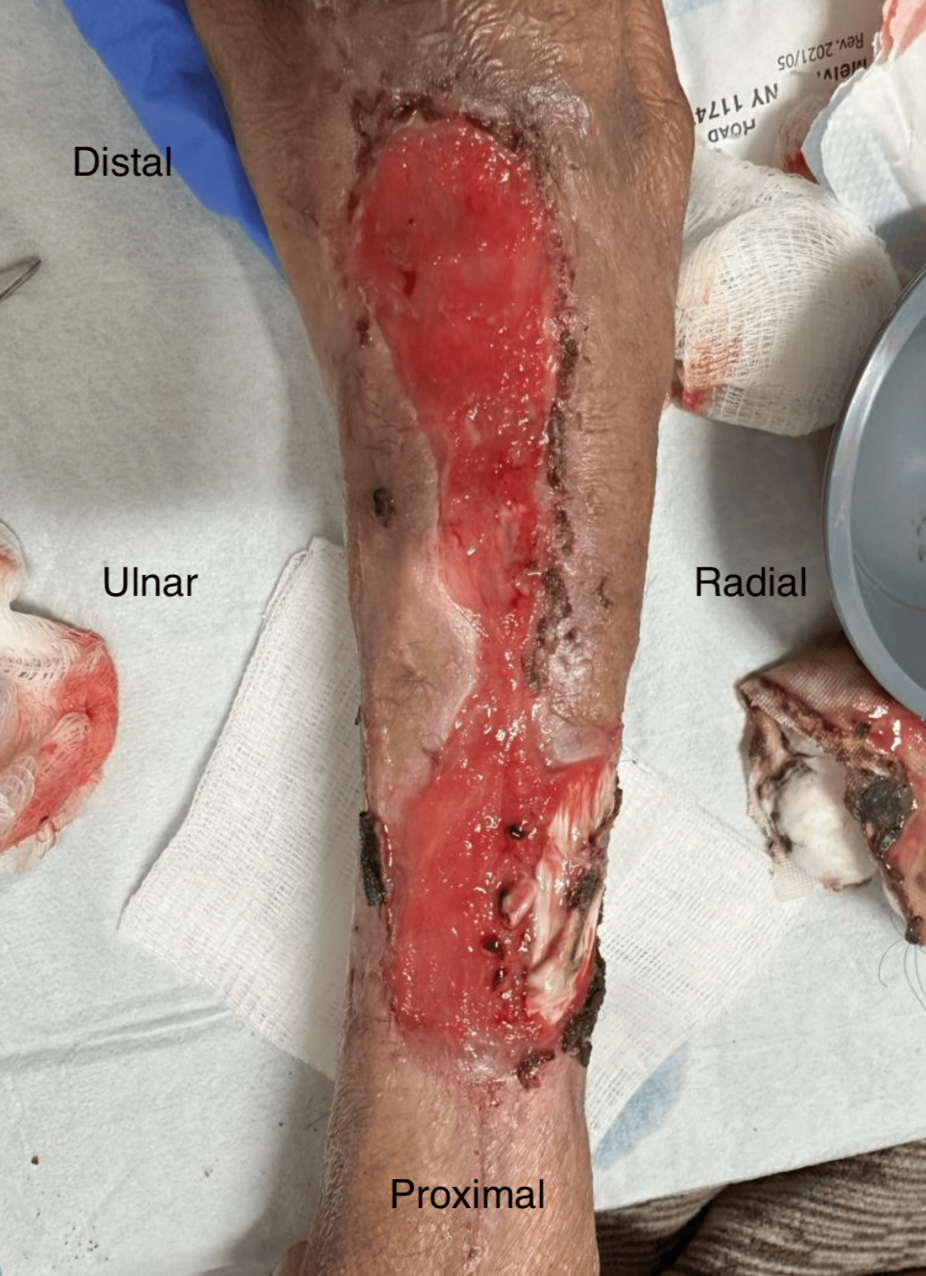
Wound depicted on 10/7/2023 measured to be 5 cm × 18cm and approximately 5 mm in depth, subsequently debrided of necrotic tendon and tissue

**Figure 2:**
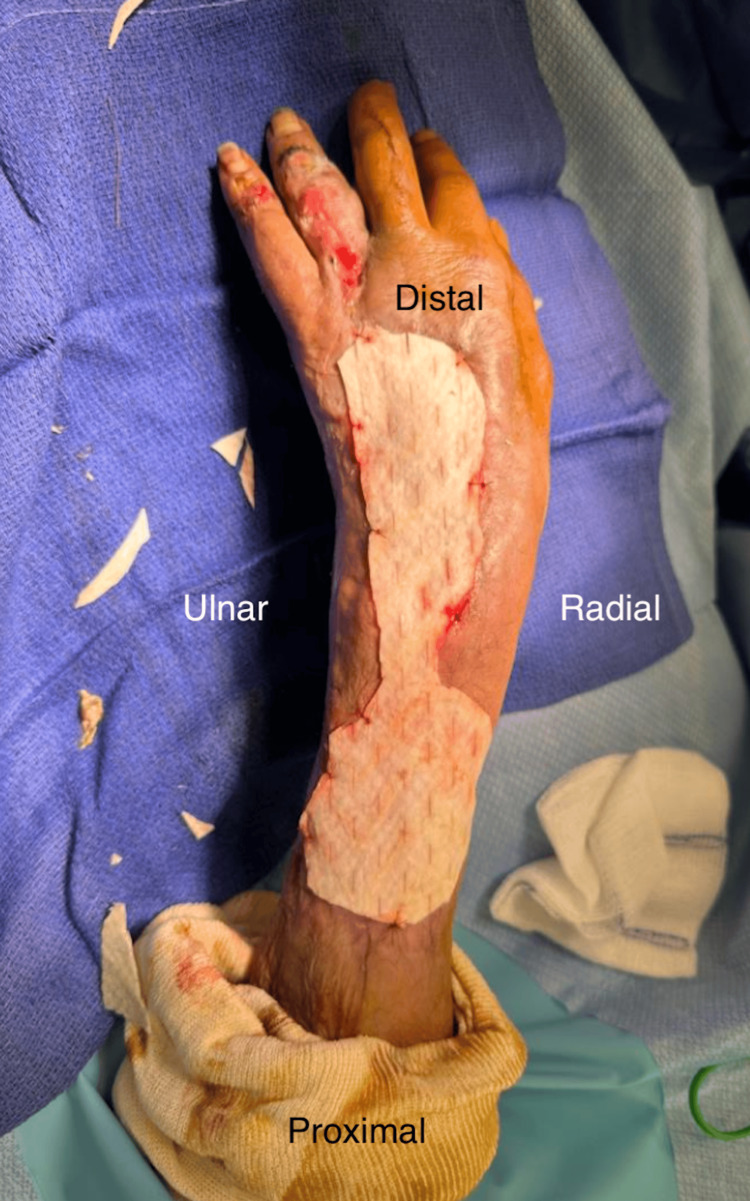
Wound after application of large Kerecis™ mesh measured at 7 cm × 20 cm

Necrotic tendon and tissue, depicted in Figure 1, necessitated aggressive debridement and multiple washouts, last recorded nine days after initial presentation, which also marked the application of a VAC negative pressure dressing set to low, intermittent suction. The large wound, measuring 5 cm × 18 cm with a depth of around 5 mm, displayed a 10% slough and exposed bone, causing significant pain rated at 7/10 by the patient. The surgical wound required further debridement of the necrotic tendon and surrounding tissue. Following this, a large mesh Kerecis^TM^ product, displayed in Figure 2, measuring 7 cm × 20 cm, was applied to facilitate healing and help act as an anti-bacterial barrier prior to any definitive management in the future.

**Figure 3:**
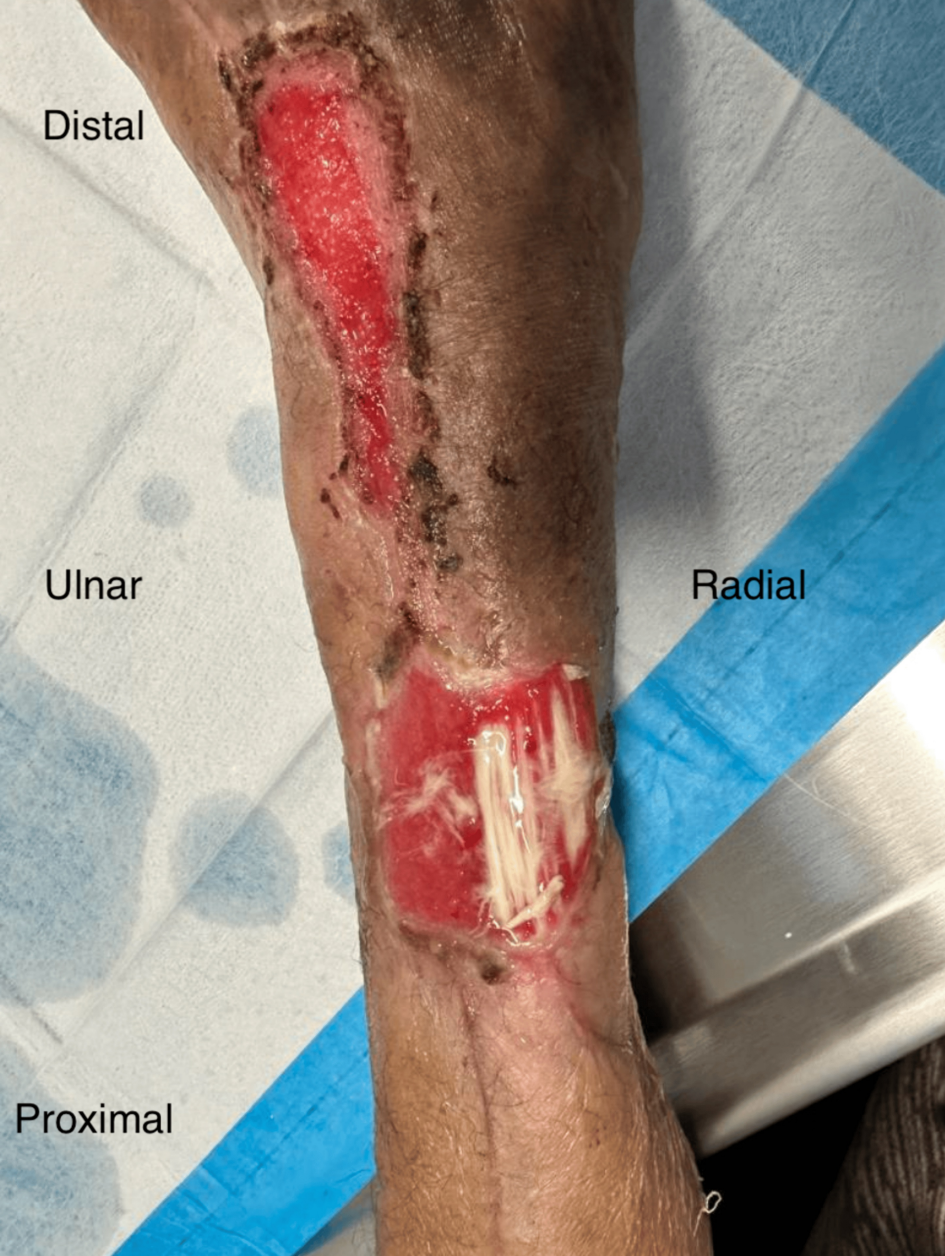
On POD 18, the wound healed in the center, creating two wounds: one distal and one proximal Tendons were still exposed but no longer necrotic, and the patient’s pain decreased to 5/10. It was noted that the wound had progressed in the periphery with epithelialization and granulation in the center.

On postoperative day (POD) 18, the wound evolved into two separate areas, with the central region showing signs of healing: epithelialization and granulation, measured as a 5 cm × 5 cm proximal and a 2 cm × 7 cm distal wound (Figure 3). The pain level had decreased to 5/10. The exposed tendon tissue was no longer necrotic. The patient was treated with silver sulfadiazine cream twice daily, a non-adherent contact layer dressing, a soft gauze wrap, and a forearm splint for immobilization of the wrist and metacarpophalangeal joints. Offloading devices and compression were used to enhance wound healing and mitigate discomfort.

**Figure 4:**
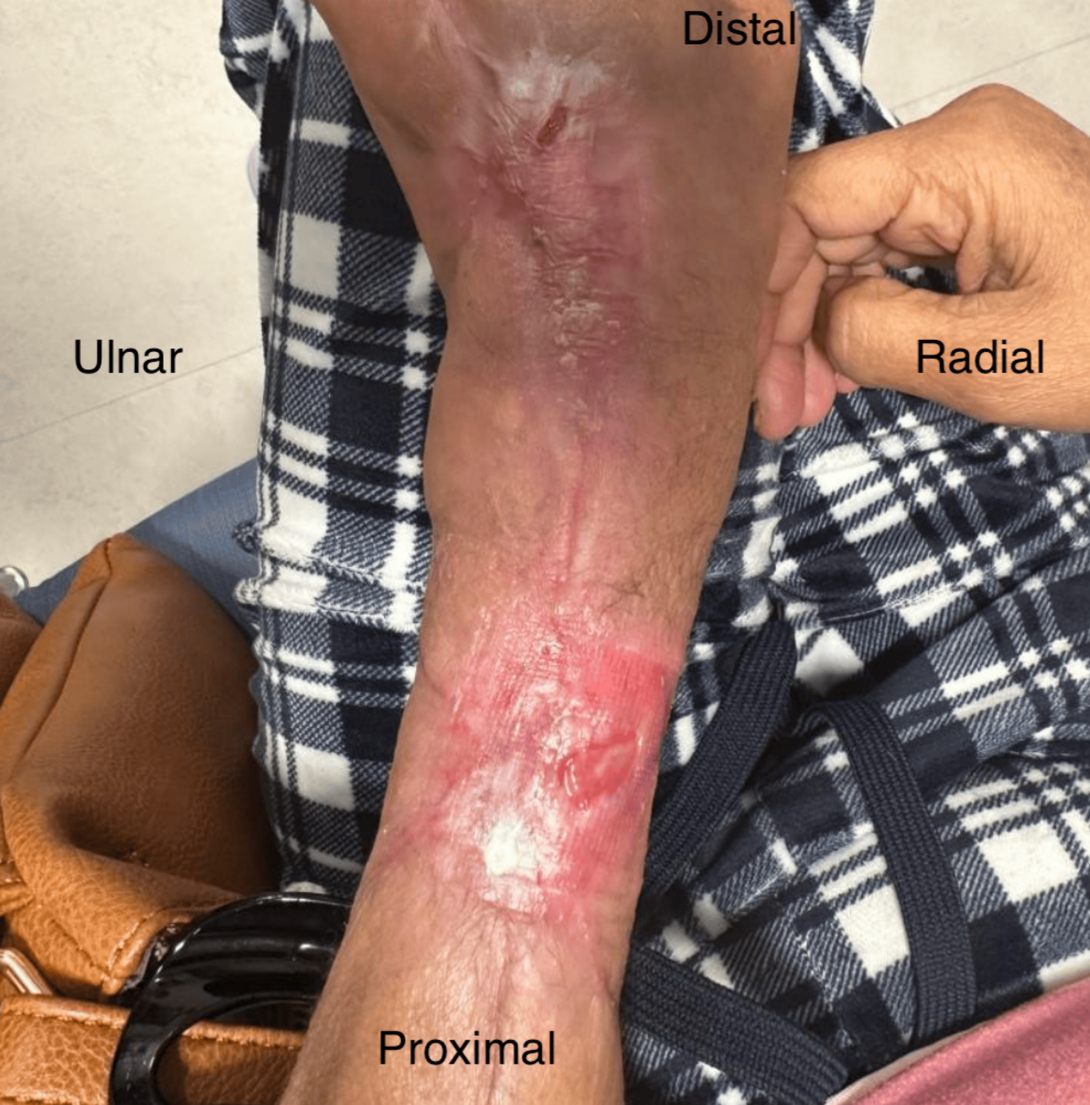
On POD 40, the wound had ceased draining, the patient was taken off antibiotics, and her pain had decreased to a 2/10

**Figure 5:**
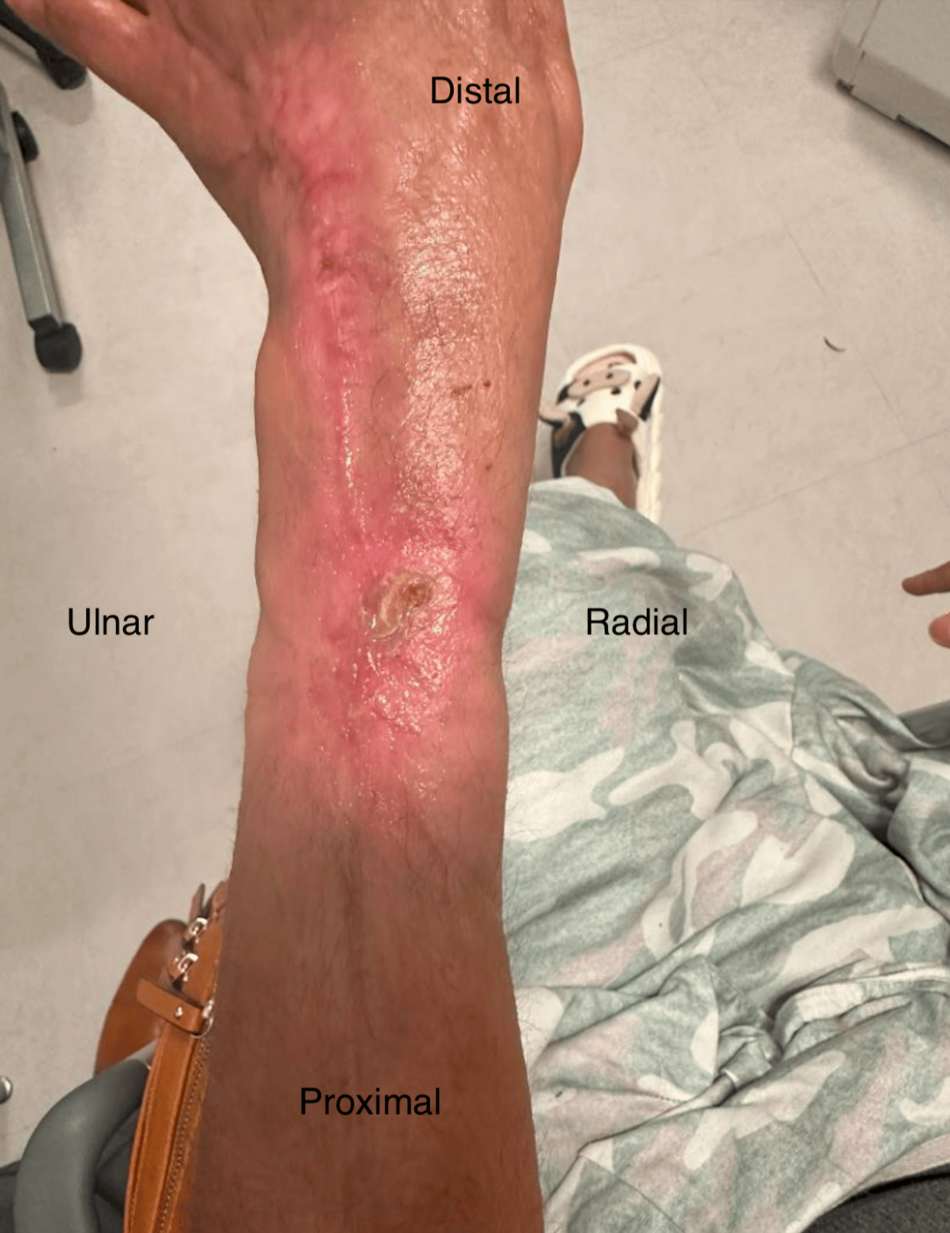
Approximately two months after the initial presentation and application of Kerecis™ (POD 74), the patient’s wound had healed fully with evident remodeling and minimal reported pain

As highlighted in Figure 4, by POD 40, the patient exhibited no drainage, was taken off all antibiotics, reported a minimal pain level of 2/10, and the splint was discontinued. This progression led to the complete wound closure by POD 74, after just a single application of the Kerecis^TM^ product without the need for secondary skin grafting, as originally planned when the product was initially placed (Figure 5).

The patient consented to the use of clinical information and the included images in this case report, and no personal health information was included to protect patient anonymity.

## Discussion

The successful treatment of a 41-year-old female with a large complex forearm wound using the Kerecis^TM^ fish skin xenograft highlights the potential of advanced biomaterials in managing large full-thickness skin defects that may not be amenable to the standard wound care strategies. This product, derived from North Atlantic Cod and traditionally used for chronic conditions such as diabetic ulcers and partial-thickness burns, has demonstrated its versatility and efficacy in acute, severe wound scenarios, including NF [7,8]. 

The structure of the Kerecis^TM^ graft, minimally processed to preserve its natural properties, closely mimics human skin. This feature is important for its effectiveness, enabling integration into human tissue, which promotes essential processes such as cellular ingrowth and neovascularization. These processes are essential to its application in situations such as a large wound due to NF, as rapid and effective wound closure is vital to prevent further infection and facilitate recovery. Moreover, the graft acts as an active scaffold, enhancing the healing process through its rich composition of omega-3 fatty acids, which are known to modulate inflammatory responses and manage cytokine storms associated with severe infections [5,9].

In this case, the patient presented with a large, full-thickness wound on the dorsal forearm and hand, complicated by exposed extensor tendons and necrotic tissue. Traditional methods, such as flap surgeries or direct skin grafts, were determined to not be feasible due to the AV fistula being within close proximity to the defect, which increased the risk of surgical complications, such as infection, bleeding, and damage to the fistula. Additionally, the exposed tendons lacked sufficient vascular support for traditional graft integration, requiring alternative methods to salvage the tendons and preserve proper extensor mechanism function. Kerecis^TM^ was chosen for its porous structure’s ability to promote vascular ingrowth and tissue regeneration, providing crucial coverage without compromising the tendons. Additionally, its anti-inflammatory properties, derived from omega-3 acids, and antimicrobial effects were leveraged to reduce local inflammation and likely contributed to the patient’s decreased pain levels, making it the optimal solution for closure in this complex wound. 

Furthermore, this case reinforces previous findings on the efficacy of fish skin xenografts, which have been demonstrated to be superior to other xenograft alternatives, such as bovine-based grafts, based on their faster wound healing time, which were 10.7 and 13.1 days, respectively [10]. One potential drawback of this newer product could be the cost associated with its application. However, despite the higher associated costs of fish skin xenografts such as Kerecis^TM^, cost-analysis studies suggest that these can be offset by the reduced overall healthcare expenditures for avoidance of secondary procedures such as skin grafting and due to faster wound healing resulting in decreased chronic wound care needs [8].

Given the promising results observed in this particular case in the upper extremity, there is a compelling need for further research and clinical trials to explore the use of Kerecis^TM^ in further wound settings. Investigating outcomes across various wound types in both the upper and lower extremities in different patient populations is important to fully understand the benefits and limitations of this treatment modality. Such studies could form the basis for its more widespread adoption in emergency and acute care settings, potentially altering the approach to wound management in patients with severe infections like NF. 

Overall, the integration of Kerecis^TM^ in this difficult case not only enhances our understanding of its capabilities but also encourages a reevaluation of current wound care practices, suggesting a shift toward more biologically active, regenerative approaches for managing severe, complex wounds. This case underlines the potential for expanding the applications of Kerecis^TM^ fish skin xenografts beyond traditional chronic wound management, indicating a significant potential to improve outcomes in wound care across a broader spectrum of clinical situations.

## Conclusions

Overall, the integration of Kerecis^TM^ in this difficult case not only enhances our understanding of its capabilities but also encourages a reevaluation of current wound care practices, suggesting a shift toward more biologically active, regenerative approaches for managing severe, complex wounds. This case underlines the potential for expanding the applications of Kerecis^TM^ fish skin xenografts beyond traditional chronic wound management, indicating a significant potential to improve outcomes in wound care across a broader spectrum of clinical situations.
